# Expression of long non-coding RNA NNT-AS1 in children with severe pneumonia and its effect on lipopolysaccharide-induced human embryonic lung fibroblast injury

**DOI:** 10.1186/s41065-026-00683-w

**Published:** 2026-05-09

**Authors:** Fanghua Jian, Jun Fu, Dan Li, Weiqiang Bao

**Affiliations:** 1 Department of Pediatrics, Longyan First Affiliated Hospital of Fujian Medical University, Longyan, 364000 China; 2 Pediatric Emergency, The Maternal and Child Health Hospital of Guangxi Zhuang Autonomous Region, Nanning, 530023 China; 3 Department of Pediatric Surgery, Pediatric Intensive Care Unit, Qianjiang Central Hospital of Chongqing, Chongqing, 409099 China; 4 Department of Pediatric, Ruian People’s Hospital, No. 108 Wansong Road, Ruian City, Wenzhou, Zhejiang 325200 China

**Keywords:** Severe pneumonia, Lipopolysaccharide, NNT-AS1, MiR-23a-3p

## Abstract

**Background:**

Severe pneumonia (SP) threatens the quality of life and well-being of children. This study aims to explore the serum NNT-AS1 expression in SP children and its impact on lipopolysaccharide (LPS)-induced injury in human embryonic lung fibroblasts.

**Methods:**

The study included 69 SP children, 62 mild pneumonia (MP) children and 75 healthy controls. A pneumonia cell model was constructed with LPS-induced MRC-5 cells. Loss-of-function experiments were performed using si-NNT-AS1 and miR-23a-3p inhibitor. The NNT-AS1 and miR-23a-3p expression was detected by real-time quantitative polymerase chain reaction. The diagnostic effect and prognostic value of NNT-AS1 were evaluated by receiver operating characteristics curves and Kaplan-Meier method. Cell viability and apoptosis were determined by 3-(4,5-dimethylthiazol-2-yl)-2,5-diphenyltetrazolium bromide (MTT) assay and flow cytometry. Inflammatory factors were measured by enzyme-linked immunosorbent assay. The interaction between NNT-AS1 and miR-23a-3p was verified by luciferase reporter gene and RNA immunoprecipitation assays. Pearson correlation coefficient was used to analyze the correlation.

**Results:**

NNT-AS1 was upregulated in SP children and showed good diagnostic efficacy (area under the curve (AUC) = 0.815). High NNT‑AS1 expression predicted a poorer survival. Silencing of NNT-AS1 increased cell viability, decreased apoptosis, and alleviated inflammatory responses. MiR-23a-3p was a target of NNT-AS1. Inhibition of miR-23a-3p partially reversed the protective effects of NNT-AS1 silencing on LPS-induced MRC-5 cell injury.

**Conclusions:**

Serum NNT-AS1 is upregulated in SP patients, and its expression is correlated with clinical prognosis. NNT-AS1 participates in LPS-induced cellular damage by regulating miR-23a-3p. These findings provide a new theoretical basis for understanding the pathogenesis of SP.

**Supplementary Information:**

The online version contains supplementary material available at 10.1186/s41065-026-00683-w.

## Background

Pneumonia is a common clinical respiratory disease and poses a major public health problem worldwide [1]. In children, pneumonia is a prevalent lung condition that may progress to severe pneumonia (SP) due to their immature immune system and relatively weak immune function [2]. In SP children, the main clinical manifestations include dyspnea, severe coughing, convulsions and persistent high fever, which can lead to death in severe cases [3, 4]. SP has become the leading cause of death in children [5]. Currently, despite advances in medical technology leading to improvements in the diagnosis and treatment of pediatric SP, the morbidity and mortality of this disease remain high [6]. Therefore, identifying novel biomarkers is of great importance, as it may provide new insights for the treatment of SP.

Long non-coding RNAs (lncRNAs) are a class of non-coding RNAs that do not encode proteins. They play critical roles in many biological processes, including gene expression regulation, cell development, and pathogenesis [7, 8]. Recently, an increasing number of studies have demonstrated that lncRNAs are involved in the pathophysiological process of pneumonia [9]. For instance, RP11-773H22.4 was upregulated in SP patients and might be involved in the pathogenesis of SP by regulating miR-1287-5p [10]. Silencing kcnq10t1 inhibited apoptosis and inflammatory responses, increased cell viability, and attenuated lipopolysaccharide (LPS)-induced injury in an LPS-induced pneumonia cell model [11]. Interestingly, NNT-AS1 is a recently identified lncRNA [12]. NNT-AS1 has been reported to be dysregulated in numerous tumor tissues [13]. In a study of pediatric refractory mycoplasma pneumonia (RMPP), Chen et al. discovered that NNT-AS1 was upregulated in RMPP patients and promoted the onset and development of RMPP [14]. In addition, NNT-AS1 exhibits important regulatory roles in tumor-related studies. NNT-AS1 was highly expressed in non-small cell lung cancer tissues and cells, and downregulation of NNT-AS1 inhibited cell function [15]. In glioma cell lines, upregulation of NNT-AS1 promoted glioma progression by targeting miR-582-5p [16]. Collectively, these studies indicate that NNT-AS1 plays a crucial regulatory role in cellular processes. However, its specific mechanism in SP remains largely unknown. Based on the above evidence, we proposed the hypothesis: serum NNT-AS1 is highly expressed in the SP children, and may exacerbate LPS-induced injury, apoptosis and inflammatory response in MRC-5 cells by targeting miR-23a-3p.

This study aims to elucidate the mechanism by which NNT-AS1 modulates LPS-induced injury in MRC-5 cells. By detecting the expression level of NNT-AS1, we evaluated its feasibility as a diagnostic biomarker for SP. Additionally, we explored the roles of NNT-AS1 and miR-23a-3p in the LPS-induced pneumocyte model. The results suggest that NNT-AS1 and miR-23a-3p are involved in the development of SP.

## Methods

### Clinical samples

This study was performed following the principles of the Declaration of Helsinki. The study was approved by the Ethics Committee of Qianjiang Central Hospital of Chongqing. Informed consent was obtained from legal guardians.

In this study, a total of 69 SP children, 62 mild pneumonia (MP) children, and 75 healthy controls were recruited from Qianjiang Central Hospital of Chongqing. In the SP group (69 SP children), the composition of pathogens was as follows: 36 cases of viral infection, 22 cases of bacterial infection, 4 cases of mycoplasma infection, and 13 cases of mixed infection. The diagnostic criteria for SP [17] primarily included respiratory failure and septic shock. Secondary criteria encompassed indicators such as respiratory rate, oxygenation index, or blood pressure. Children were diagnosed with SP if they met at least one major criterion or all three minor criteria. Inclusion criteria for SP children: (1) met the diagnostic criteria for SP, (2) all subjects had not received any treatment prior to blood collection, (3) all subjects excluded complications such as other infections or immune disorders, and (4) complete clinical data. All pediatric children received standard symptomatic treatment, including vital sign monitoring, nutritional support, oxygen therapy, and antibiotic treatment. Baseline characteristics including sex, age, hospital stay days, respiratory complications, cardiovascular complications, digestive complications, premature birth, congenital heart disease, white blood cell (WBC), neutrophil (NEU), platelet (PLT), C-reactive protein (CRP), lactate dehydrogenase (LDH), procalcitonin (PCT), D-Dimer (DD), partial pressure of arterial oxygen/fraction of inspired oxygen (PaO_2_/FiO_2_) and respiratory support were summarized in Table 1. Venous blood were collected from all participants on an empty stomach the day after admission. Serum was separated and stored at -80 °C for subsequent analysis.

**Table 1:** Comparison of baseline data for subjects

Parameters	Severe pneumonia (*n* = 69)	Mild pneumonia (*n* = 62)	Control (*n* = 75)	*P*_1_ value	*P*_2_ value	*P*_3_ value
Sex (male/female)	36/33	30/32	40/30	0.984	0.857	0.906
Age (years)	5.25±1.58	5.66±1.41	5.25±1.44	1.000	0.186	0.312
Hospital stay (days)	11.91±3.41	5.52±1.98				< 0.001
Respiratory complications	13	4				0.035
Cardiovascular complications	10	5				0.249
Digestive complications	2	1				0.623
Premature birth	11	4				0.089
Congenital heart disease	9	4				0.208
WBC (× 10^9^/L)	8.44±3.79	8.92±2.75	8.21±1.59	0.880	0.230	0.729
NEU (× 10^9^/L)	4.76±2.02	4.88±1.63	4.33±1.02	0.249	**0.039**	0.926
PLT (× 10^9^/L)	313.98±72.36	306.55±82.20	214.93±54.65	**< 0.001**	**< 0.001**	0.859
CRP (mg/mL)	31.29±7.84	8.18±4.40	4.01±0.53	**< 0.001**	**< 0.001**	< 0.001
LDH (U/L)	346.26±83.65	305.88±71.96	254.29±45.36	**< 0.001**	**< 0.001**	0.006
PCT (ng/mL)	0.15±0.07	0.10±0.05	0.08±0.04	**< 0.001**	**0.028**	< 0.001
DD (µg/mL)	1.29±0.33	0.30±0.13	0.19±0.09	**< 0.001**	**< 0.001**	< 0.001
PaO_2_/FiO_2_ (mmHg)	228.57 ± 42.69	358.74 ± 59.76	426.92 ± 82.80	**< 0.001**	**< 0.001**	< 0.001
Respiratory support						
Non-invasive ventilation	15					
Invasive ventilation	14					

*Control* healthy individuals, *WBC* white blood cell, *NEU* neutrophil, *PLT* platelet, *CRP* C-reactive protein, *LDH* lactate dehydrogenase, *PCT* Procalcitonin, D-Dimer: DD, PaO_2_/FiO_2_ : partial pressure of arterial oxygen/fraction of inspired oxygen, *P*_1_ severe pneumonia vs. control group, *P*_2_ mild pneumonia vs. control group, *P*_3_ severe pneumonia vs. mild pneumonia group, *P* < 0.05 indicates a significant difference

### Follow-up

SP children were followed up for 28 days. The endpoint was death or the last follow-up visit, and survival status was recorded accordingly.

### Cell culture and modeling of pneumonia cells

MRC-5 cells were obtained from the Shanghai Cell Bank. The cells were cultivated in Dulbecco’s Modified Eagle Medium (DMEM, Sigma-Aldrich, Saint Louis, MO, USA) supplemented with 10% fetal bovine serum (FBS, Biowest, Nuaille, FRA) and 1% penicillin-streptomycin (Invitrogen, Carlsbad, CA, USA). Cells were maintained in a 37 °C incubator (Thermo Fisher Scientific, Waltham, MA, USA) with 5% CO_2_. In order to construct a pneumonia cell model, MRC-5 cells were treated with LPS (Sigma-Aldrich) at various concentrations (0, 5, 10, and 15 µg/mL) for 12 h. In addition, poly(I:C) (Sigma-Aldrich) was added directly to MRC-5 cell culture medium to a final concentration of 10 µg/mL, and the cells were incubated for 24 h [18].

### Cell transfection

Small interfering negative control (si-NC), si-NNT-AS1, inhibitor/mimic-NC, and miR-23a-3p inhibitor/mimic were purchased from RiboBio (Guangzhou, CHN). According to the product manuals, plasmids and oligonucleotides were introduced into LPS-induced MRC-5 cells using Lipofectamine 2000 (Invitrogen) transfection reagent. After 24 h of incubation, the cells were harvested for subsequent procedures.

### RNA isolation and real-time quantitative polymerase chain reaction (RT-qPCR)

The total RNA from serum and cells was extracted using TRIzol reagent (Invitrogen). RNA quality and purity were assessed using an ultraviolet spectrophotometer (Thermo Fisher Scientific). Only samples with OD260/OD280 values between 1.8 and 2.0 were used. In accordance with the manufacturer’s protocol, remaining genomic DNA was removed from total RNA using the PrimeScript RT Reagent Kit (Takara, Tokyo, Japan). Complementary DNA was synthesized through reverse transcription. RT-qPCR was performed using SYBR Premix Ex Taq II (Takara) following strict steps. The thermal cycling conditions: 95 °C for 10 s followed by 40 cycles of amplification (95 °C for 5 s, 60 °C for 15 s, 72 °C for 10 s) and dissociation curve analysis. Expression levels of lncRNA and miRNA were calculated using the 2^−ΔΔCt^ method, with GAPDH and U6 as internal references. The primer sequences are shown in Table S1.

### Cell viability

3-(4,5-dimethylthiazol-2-yl)-2,5-diphenyltetrazolium bromide (MTT) assay was utilized to assess the cell viability of LPS-induced MRC-5 cells after transfection. Cells were cultured in 96-well plates (Corning, NY, USA). Then, 20 µL of 5 mg/mL MTT solution (Aladdin, Shanghai, CHN) was added to each well followed by incubation for 4 h. Subsequently, 150 µL of dimethyl sulfoxide (DMSO, Sigma-Aldrich) was added to each well, and the plates were gently shaken for 15 min to guarantee complete dissolution of formazan particles. Finally, absorbance was measured using a microplate reader (Thermo Fisher Scientific).

### Cell apoptosis

Annexin V-fluorescein isothiocyanate (FITC) and propidium iodide (PI) double-staining approach (BD Biosciences, San Diego, CA, USA) was utilized to measure apoptosis in LPS-induced MRC-5 cells. Cells at 80% confluence were plated into 6-well plates at a density of 5 × 10^4^ for the transfection. Cells were collected and washed with pre-cooled phosphate buffered saline (PBS, Solarbio, Beijing, CHN). Next, 5 µL of FITC and 5 µL of PI were added sequentially, and the mixture was gently mixed. Finally, cellular apoptosis was analyzed by flow cytometry (FACScalibur, BD Biosciences).

### Enzyme-linked immunosorbent assay (ELISA)

Cellular interleukin (IL)-6, IL-8 and IL-10 levels were quantified by ELISA. The detection procedures were performed strictly according to the guidelines of the human IL-6, IL-8, and IL-10 ELISA kits (Solarbio). A microplate reader was used to measure the absorbance.

### Dual luciferase reporter gene assay

The target miRNAs of NNT-AS1 were predicted using the Starbase online database. Wild-type (WT)-NNT-AS1 and Mutant-type (MUT)-NNT-AS1 luciferase plasmids were constructed based on the NNT-AS1 sequence. Cells were transfected with Lipofectamine 2000 reagent following the manufacturer’s protocol and incubated for 24 h. Luciferase activity was determined using a dual luciferase reporter system (Promega, Madison, WI, USA).

### RNA immunoprecipitation (RIP)

An RIP kit (Absin, Shanghai, CHN) was employed to conduct the experiment. Cells were harvested and rinsed with pre-chilled PBS. Subsequently, the cells were lysed on ice using the RIP lysis buffer. The magnetic beads were incubated with anti-immunoglobulin G (IgG, Sigma-Aldrich) or anti-Argonaute 2 (Ago2, Sigma-Aldrich) at 4 °C overnight. After incubation, the beads were washed with pre-chilled buffer, and the supernatant was removed. Finally, RNA was isolated for analysis.

### Bioinformatics

The potential target genes of miR-23a-3p were predicted using the online databases TargetScan, miRWalk, and EVmiRNA. The overlapping target genes were presented in a Venn diagram and selected for further analysis. Gene ontology (GO) and kyoto encyclopedia of genes and genomes (KEGG) analyses of the overlapping target genes were performed using the Database for Annotation, Visualization and Integrated Discovery (DAVID).

### Statistical analysis

Data were analyzed using GraphPad Prism 8 (GraphPad Software, San Diego, CA, USA) and SPSS 23.0 (SPSS Inc., Chicago, IL, USA). All in vitro experiments were performed with at least three independent biological replicates and three technical replicates. The statistical data were presented as the mean ± standard deviation. One-way analysis of variance (ANOVA) was used for comparisons among multiple groups, and Student’s t-test was used for two-group comparisons. The diagnostic value of NNT-AS1 was assessed using receiver operating characteristic (ROC) curves. Kaplan-Meier method was utilized to examine the association between NNT-AS1 expression and overall survival. A Cox regression model was used to identify independent prognostic factors in SP children. Pearson correlation coefficient was utilized to determine the relationship between NNT-AS1 and miR-23a-3p. All statistical tests were two-sided. *P* < 0.05 was regarded as statistically significant.

## Results

### Comparison of baseline data

Table 1 showed the comparison of clinical data among SP children, MP children, and the healthy controls. After statistical analysis, no significant differences were detected in sex, age, and WBC count among the three groups (*P* > 0.05). Compared with the healthy control group, PLT, CRP, LDH, PCT, and DD levels were significantly elevated, while PaO_2_/FiO_2_ levels were significantly reduced in SP children. In MP children, NEU, PLT, CRP, LDH, PCT, and DD levels were also significantly upregulated, and PaO_2_/FiO_2_ levels were significantly reduced (*P* < 0.05). Furthermore, significant differences in hospital stay duration, respiratory complications, CRP, LDH, PCT, DD, and PaO_2_/FiO_2_ levels were observed between MP and SP children (*P* < 0.05). In the SP children group, 15 children required non-invasive ventilation, and 14 children required invasive ventilation.

### Diagnostic and prognostic value of NNT-AS1 in SP children

**Fig. 1:**
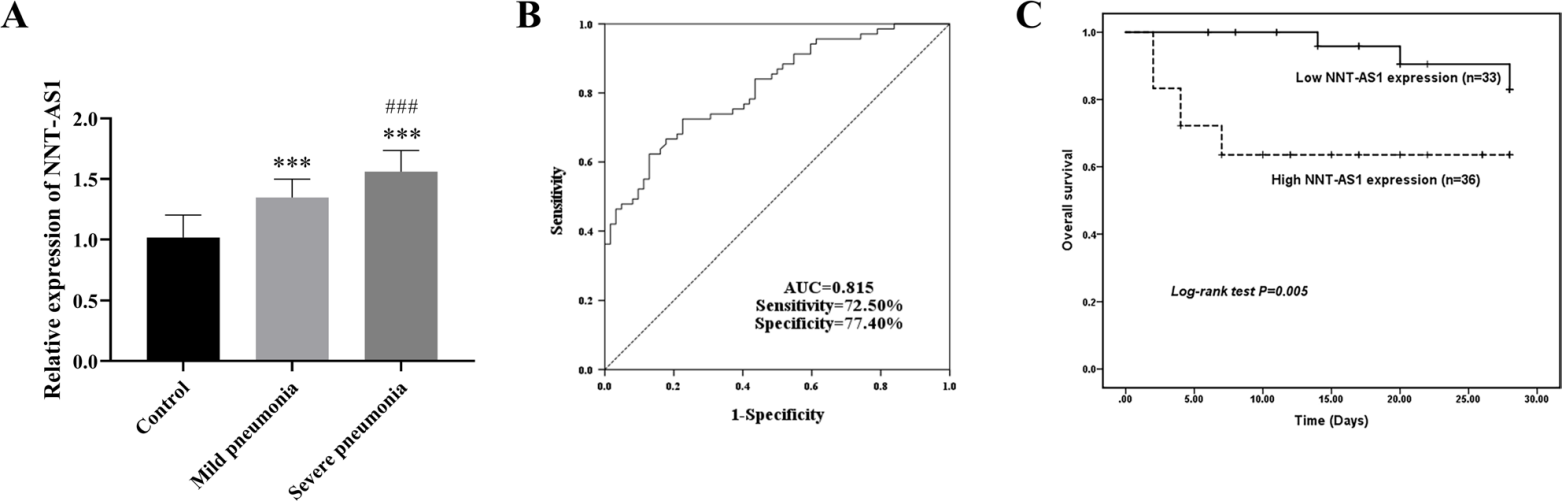
Clinical value of serum NNT-AS1 in SP children. **(A)**. Serum NNT-AS1 levels in the three groups. **(B)**. ROC curve indicating the diagnostic value of serum NNT-AS1 for SP. **(C)** Overall survival analysis was performed using the Kaplan-Meier method. Statistical significance was determined using one-way ANOVA followed by Tukey’s multiple comparisons test. (^***^P < 0.001 vs. Control; ^###^*P* < 0.001 vs. Mild pneumonia)

To further explore the function of NNT-AS1 in pediatric pneumonia, serum NNT-AS1 expression was measured by RT-qPCR. As presented in Fig. 1A, serum NNT-AS1 expression was elevated in MP and SP children, and a significant difference was also observed between the MP and SP groups (*P* < 0.001). Serum NNT-AS1 effectively distinguished SP children from healthy controls, with an area under the curve (AUC) of 0.815, a sensitivity of 72.50%, and a specificity of 77.40% (Fig. 1B). Based on the mean serum NNT-AS1 levels in SP children, children were divided into a high-NNT-AS1-expression group (*n* = 36) and a low-NNT-AS1-expression group (*n* = 33). Children with low NNT-AS1 expression had a better overall survival (*P* < 0.05, Fig. 1C). Moreover, univariate analysis (Table 2) revealed that NNT-AS1, respiratory system, CRP and PaO_2_/FiO_2_ were associated with overall survival. Multivariate analysis demonstrated that NNT-AS1 was an independent risk factor for poor prognosis in SP children.

**Table 2:** Cox regression analysis of clinicopathological variables for overall survival of severe pneumonia in children

Variables	Univariate analysis		Multivariate analysis	
	HR (95% CI)	*P* value	HR (95% CI)	*P* value
Sex (male/female)	1.051 (0.391–2.823)	0.921	/	/
Age (years)	2.757 (0.957–7.940)	0.060	/	/
Hospital of stay (days)	0.445 (0.143–1.382)	0.161	/	/
Respiratory complications	2.904 (1.050–8.018)	**0.040**	2.764 (0.911–8.389)	0.073
Cardiovascular complications	2.813 (0.976–8.108)	0.055	/	/
Digestive complications	2.094 (0.275–15.949)	0.476	/	/
Premature birth	1.893 (0.600-5.782)	0.282	/	/
Congenital heart disease	2.239 (0.722–6.946)	0.163	/	/
WBC (× 10^9^/L)	1.329 (0.498–3.547)	0.570	/	/
NEU (× 10^9^/L)	0.565 (0.196–1.627)	0.290	/	/
PLT (× 10^9^/L)	1.203 (0.451–3.207)	0.712	/	/
CRP (mg/mL)	4.373 (1.244–15.376)	**0.021**	2.897 (0.779–10.770)	0.112
LDH (U/L)	0.909 (0.337–2.447)	0.850	/	/
PCT (ng/mL)	0.640(0.222–1.848)	0.410	/	/
DD (µg/mL)	0.989(0.367–2.667)	0.983	/	/
PaO_2_/FiO_2_ (mmHg)	3.130 (1.009–9.709)	**0.048**	1.605 (0.465–5.540)	0.454
Respiratory support	1.313 (0.741–2.328)	0.351	/	/
NNT-AS1	4.924 (1.396–17.369)	**0.013**	4.375 (1.143–16.747)	**0.031**

*HR* hazard ratio, 95% *CI* 95% confidence interval, *WBC* white blood cell, *NEU* neutrophil, *PLT* platelet, *CRP* C-reactive protein, LDH lactate dehydrogenase, *PCT* Procalcitonin, D-Dimer: DD, PaO_2_/FiO_2_: partial pressure of arterial oxygen/fraction of inspired oxygen, *P* < 0.05 indicates a significant difference

### Effect of NNT-AS1 on related indexes in LPS-induced MRC-5 cells

**Fig. 2:**
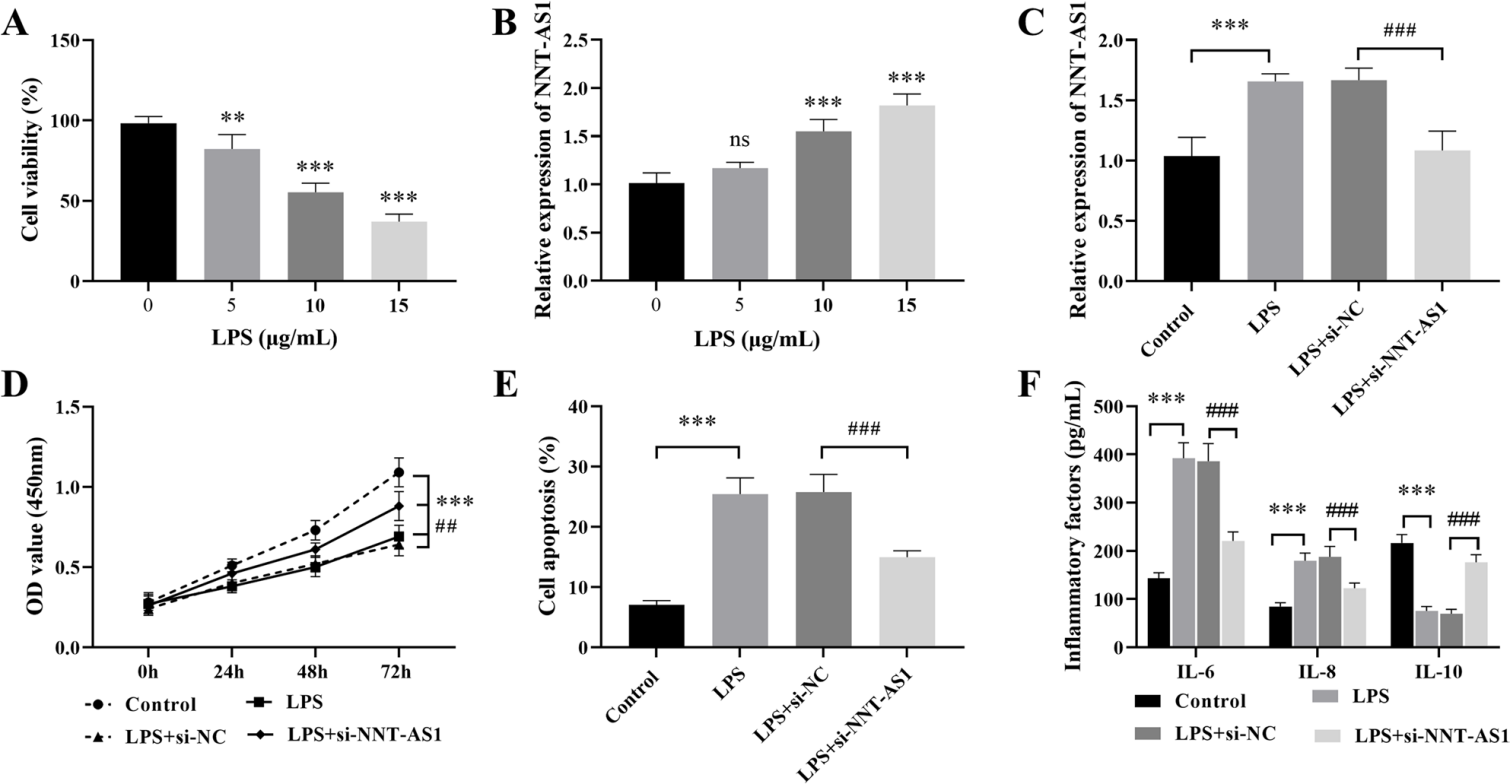
Effects of NNT-AS1 on LPS-induced injury in MRC-5 cells. Cell viability **(A)** and NNT-AS1 levels **(B)** in MRC-5 cells treated with increasing concentrations of LPS. Effects of si-NNT-AS1 transfection on NNT-AS1 levels **(C)**, cell viability **(D)**, apoptosis **(E)**, and inflammatory factors **(F)**. Data were analyzed by one-way ANOVA followed by Tukey’s multiple comparisons test. (ns *P* > 0.05, ^**^P < 0.01, ^***^P < 0.001 vs. Control; ^##^*P* < 0.01, ^###^*P* < 0.001 vs. LPS + si-NC)

In this research, MRC-5 cells were exposed to different concentrations of LPS to simulate lung fibroblast injury in SP children in vitro. As depicted in Fig. 2A, as the LPS concentration increased, cell viability significantly decreased (*P* < 0.01), confirming the successful establishment of the pneumonia cell model. Moreover, as the LPS concentration increased, NNT-AS1 levels also increased (*P* < 0.001, Fig. 2B). Based on these findings, 15 µg/mL was selected as the induction concentration of LPS and was used for subsequent experiments. Transfection efficiency was confirmed by RT‑qPCR. Compared with the si‑NC group, NNT‑AS1 expression was significantly decreased in the si‑NNT‑AS1 group (*P* < 0.001, Figure S1A). Moreover, LPS treatment increased NNT-AS1 expression, and silencing NNT-AS1 effectively reversed this effect (*P* < 0.001, Fig. 2C). LPS treatment decreased cell viability and increased apoptosis in MRC-5 cells, whereas transfection with si-NNT-AS1 increased cell viability and decreased apoptosis (*P* < 0.01, Fig. 2D-E). Silencing NNT-AS1 effectively reversed LPS-induced inflammation responses by decreasing IL-6 and IL-8 levels and increased IL-10 levels (*P* < 0.001, Fig. 2F). In order to deeply explore the role of NNT-AS1 in the virus-induced pneumonia model, this study further employed poly(I: C) to infect MRC-5 cells. The results showed that the expression levels of NNT-AS1, the changes in cell viability, cell apoptosis, and inflammatory responses were consistent with those observed in the LPS-induced cell injury model (*P* < 0.01, Figure S2A-D). Overall, these results suggest that silencing NNT-AS1 significantly alleviates the cell damage induced by LPS and viral mimics.

### Interaction of NNT-AS1 with miR-23a-3p

**Fig. 3:**
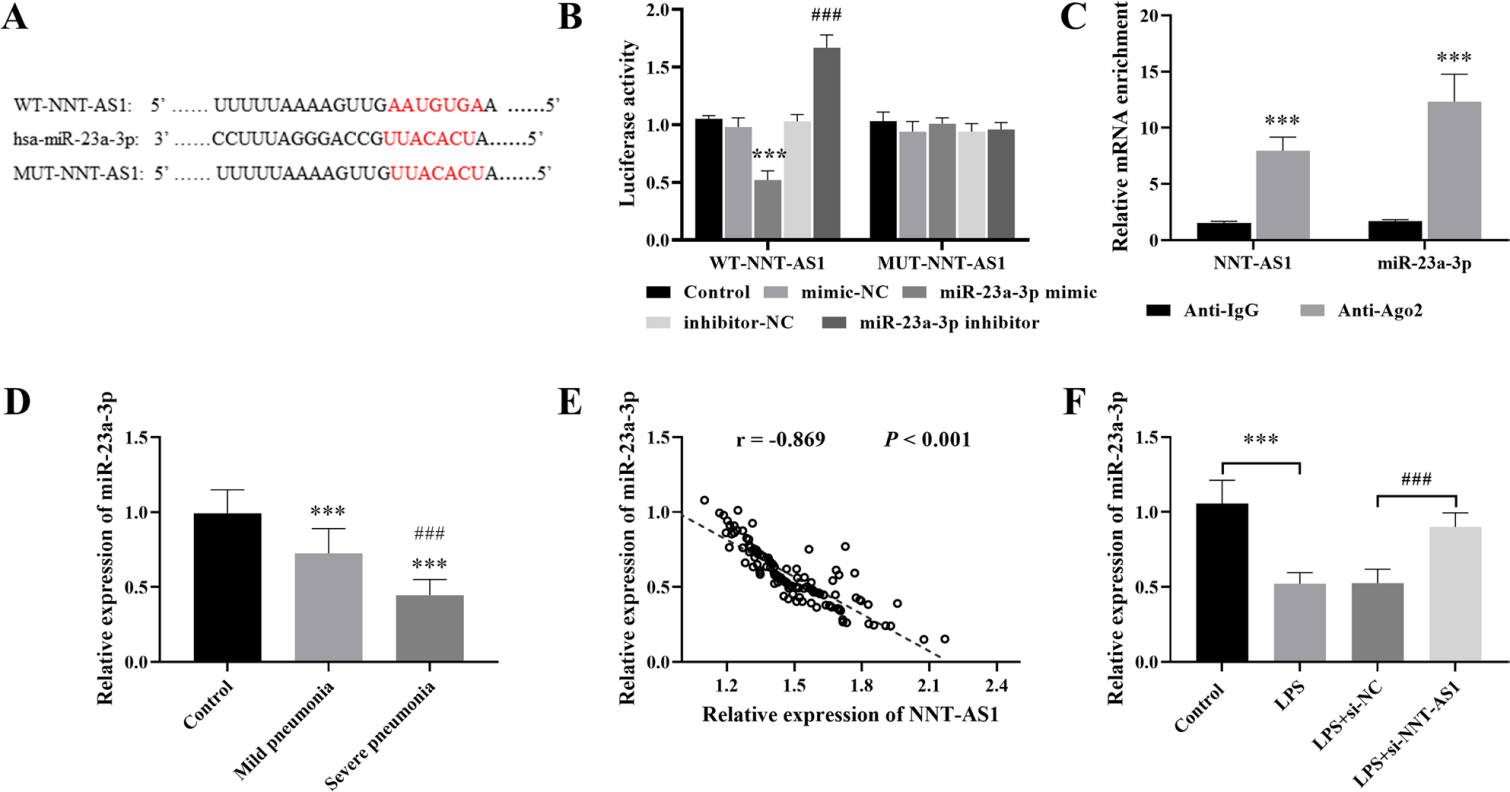
MiR-23a-3p is a target of NNT-AS1. **(A)**. Complementary binding sequences between NNT-AS1 and miR-23a-3p. Dual luciferase reporter gene assay **(B)** and RIP assay **(C)** validated the interaction. **(D)**. Serum miR-23a-3p levels in the three groups. **(E)**. Correlation analysis between NNT-AS1 and miR-23a-3p. **(F)**. Effect of si-NNT-AS1 transfection on cellular miR-23a-3p expression. Statistical significance was assessed using Student’s t-test or one-way ANOVA followed by Tukey’s multiple comparisons test. (^***^P < 0.01 vs. mimic-NC, ^***^P < 0.001 vs. Control; ^###^*P* < 0.01 vs. inhibitor-NC, ^###^*P* < 0.001 vs. Mild pneumonia, ^###^*P* < 0.001 vs. LPS + si-NC)

To further explore the molecular mechanism of NNT-AS1, the Starbase online database was used to predict the target miRNA miR-23a-3p, which possesses complementary binding sites with NNT-AS1 (Fig. 3A). In cells transfected with WT-NNT-AS1, luciferase activity was reduced by miR-23a-3p mimic but increased by miR-23a-3p inhibitor (*P* < 0.001, Fig. 3B). Meanwhile, RIP assay results indicated that the enrichment of NNT-AS1 and miR-23a-3p was higher in the anti-Ago2 group (*P* < 0.001, Fig. 3C). Compared with the control group, serum miR-23a-3p levels were downregulated in MP and SP children, with significant differences also observed between the MP and SP groups (*P* < 0.001, Fig. 3D). NNT-AS1 was significantly negatively correlated with miR-23a-3p in SP children (*r*=-0.869, *P* < 0.001, Fig. 3E). Subsequently, the regulatory impact of NNT-AS1 on miR-23a-3p was examined in LPS-induced cells. MiR‑23a‑3p expression was significantly downregulated in the miR‑23a‑3p inhibitor group (*P* < 0.001, Figure S1B). Moreover, LPS treatment downregulated miR-23a-3p expression, whereas silencing NNT-AS1 effectively reversed this effect (*P* < 0.001, Fig. 3F). Collectively, these results suggest that NNT-AS1 interacts with miR-23a-3p.

### Effect of miR-23a-3p on relevant indicators in LPS-induced MRC-5 cells

**Fig. 4:**
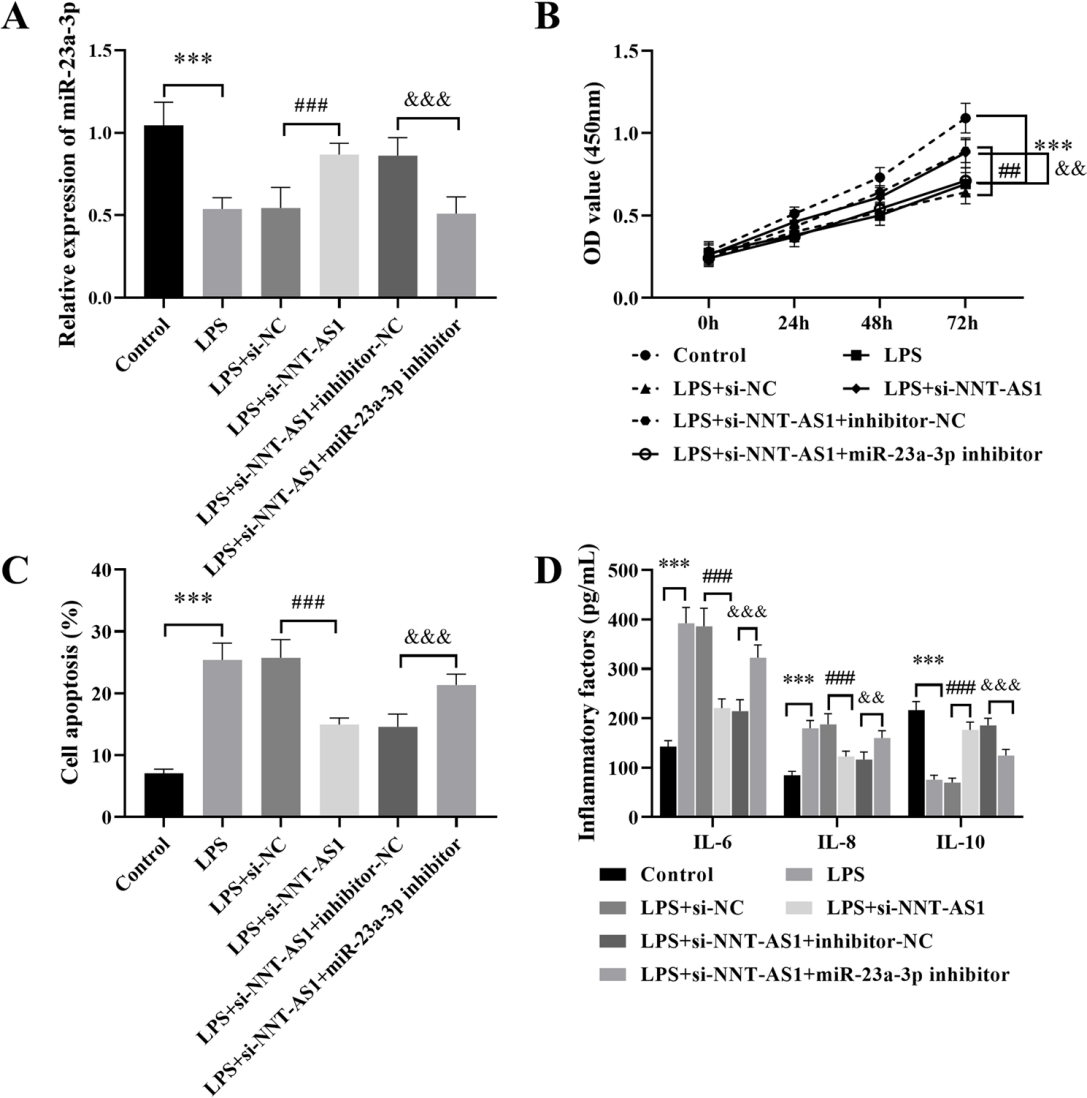
Effects of miR-23a-3p on LPS-induced MRC-5 cells. Effects of co-transfection of si-NNT-AS1 and miR-23a-3p inhibitor on miR-23a-3p **(A)**, cell viability **(B)**, apoptosis **(C)**, and inflammatory factors **(D)**. Data were analyzed by one-way ANOVA followed by Tukey’s multiple comparisons test. (^***^P < 0.001 vs. Control; ^##^*P* < 0.01, ^###^*P* < 0.001 vs. LPS + si-NC; ^&&^*P* < 0.01, ^&&&^*P* < 0.001 vs. LPS + si-NNT-AS1 + inhibitor-NC)

Subsequent findings indicated that inhibition of miR-23a-3p reversed the increased cell viability and decreased apoptosis induced by silencing NNT-AS1 (*P* < 0.01, Fig. 4A-C). Similarly, the cellular levels of IL-6, IL-8 and IL-10 were also partially reversed (*P* < 0.01, Fig. 4D). Collectively, NNT-AS1 modulates LPS-induced cell injury by regulating miR-23a-3p expression.

### Prediction of miR-23a-3p target genes

**Fig. 5:**
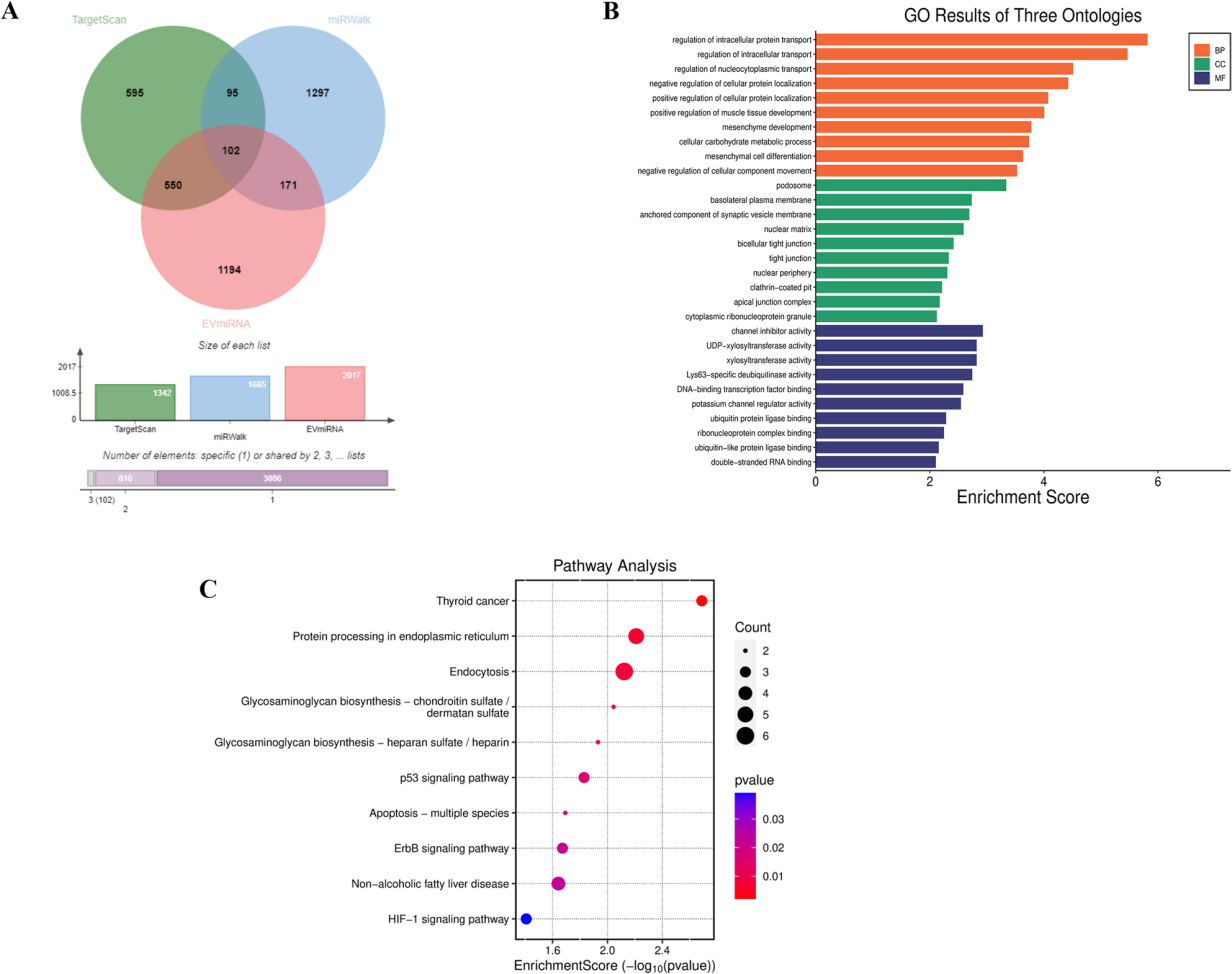
Prediction and functional analysis of miR-23a-3p target genes. **(A)**. Venn displays predicted target genes. **(B)**. GO enrichment analysis. **(C)**. KEGG pathway analysis

Three online databases were used to predict the potential target genes of miR-23a-3p. A total of 102 overlapping target genes were identified using a Venn diagram (Fig. 5A). These overlapping target genes were then subjected to GO and KEGG analyses. As shown in Fig. 5B, for biological processes (BP), the target genes were mainly enriched in regulation of intracellular protein transport, regulation of intracellular transport, and regulation of nucleocytoplasmic transport. For cellular component (CC), these genes were mainly enriched in podosome, basolateral, and membrane. Regarding molecular function (MF), they were primarily associated with channel inhibitor activity, UDP-xylosyltransferase activity, and xylosyltransferase activity. The KEGG analysis indicated that these target genes were mainly enriched in signaling pathways including thyroid cancer, protein processing in endoplasmic reticulum, and endocytosis (Fig. 5C).

## Discussion

The state of children with SP progresses rapidly and is extremely difficult to treat. It has long been a pivotal and arduous problem in pediatrics, presenting a serious threat to the life and well-being of children [19]. At present, the clinical diagnosis of childhood SP mainly depends on imaging examinations and blood routine tests. However, these diagnostic methods have limitations regarding timeliness and precision [20]. Therefore, identifying a reliable biomarker may provide new insights into the diagnosis and prognosis of SP.

Prior studies have indicated that the inflammatory response plays a pivotal role in the onset and progression of SP. When the body is exposed to external stimuli, cytokines and inflammatory factors are highly expressed, which further exacerbates the inflammatory response and ultimately contributes to the development of SP [21]. In the present study, silencing NNT-AS1 partially reversed the LPS-induced enhancement of the inflammatory response in MRC-5 cells. Additionally, the levels of IL-6, IL-8 and IL-10 were partially reversed following co-transfection with si-NNT-AS1 and miR-23a-3p inhibitor.

While MRC-5 cells are not the primary responsive cells in acute pneumonia, they play an important role in the development of lung inflammation [22]. Following lung epithelial damage, MRC-5 cells become activated and secrete abundant cytokines and chemokines, including transforming growth factor-beta (TGF-β) and IL-6. These secreted factors regulate the local immune response and impact the repair of epithelial cells [23]. Conversely, the abnormal immune response caused by immune dysregulation acts on MRC-5 cells, inducing their transdifferentiation into myofibroblasts, which promotes tissue fibrosis and impairs lung structure and function [24].

Currently, several studies have confirmed that lncRNAs are closely associated with the development of pneumonia and are involved in LPS-induced damage in human embryonic lung fibroblasts [25]. In-depth exploration of the expression characteristics and regulatory mechanisms of lncRNAs may provide novel strategies for the treatment of SP. According to the published relevant studies, NNT-AS1 is aberrantly expressed in various tumor tissues or cells and promotes cell proliferation and migration [26]. Yao et al. indicated that NNT-AS1 levels were increased in prostate cancer cells, and knockdown of NNT-AS1 suppressed malignant cell function [27]. Although these studies focused on tumorigenesis, they support the notion that NNT-AS1 exerts critical regulatory roles in cellular processes. This prompted us to explore its function in LPS-induced injury in human embryonic lung fibroblasts. In this study, we discovered that silencing NNT-AS1 remarkably attenuated LPS-induced cell death and inflammatory injury. These findings strongly indicate that silencing NNT-AS1 alleviates LPS-induced injury in human embryonic lung fibroblasts.

It is well known that lncRNA can act as miRNA sponges to participate in disease progression [28]. This study verified that miR-23a-3p is a target miRNA of NNT-AS1. MiR-23a-3p has been reported to serve important roles in various diseases. For instance, its expression was down-regulated in patients with acute kidney injury and HK-2 cells, whereas overexpression of miR-23a-3p alleviated LPS-induced proliferation inhibition, apoptosis and cytokine upregulation in HK-2 cells [29]. However, the role of miR-23a-3p in SP remains largely unclear. In this study, miR-23a-3p was downregulated in the serum and MRC-5 cells, and silencing NNT-AS1 effectively reversed this downregulation. Additionally, inhibition of miR-23a-3p partly abrogated the impact of NNT-AS1 on LPS-induced cell damage. These results suggest that miR-23a-3p exerts a protective effect in LPS-induced lung fibroblast injury.

The current study has some limitations. Firstly, the samples were obtained from a single hospital with a relatively small sample size, which may affect the generalizability of the results. Secondly, children are younger and their immune systems are not yet fully developed. Moreover, some children have underlying diseases and are suffering from mixed infections. These factors combined result in a relatively higher mortality rate among children with SP. Thirdly, the LPS-induced MRC-5 cell model mainly simulates the inflammatory damage caused by Gram-negative bacteria. Given that viral infections and mycoplasma pneumonia dominate in clinical cohort, the direct correlation between this in vitro model and the clinical pathogenesis is limited. Fourthly, the in vitro lung fibroblast model cannot fully replicate the complex pathological processes in vivo, which may slightly bias the results. Finally, the predicted downstream targets of miR-23a-3p were only analyzed bioinformatically, without further validation at the mRNA or protein level, and rescue experiments were not performed, making it difficult to clarify their regulatory relationships and functional links. Future studies should expand the source and volume of samples, and use viral (e.g., RSV, influenza virus) or mycoplasma antigens to stimulate MRC-5 cells in order to validate the mechanism of action of the NNT-AS1/miR-23a-3p axis. Furthermore, should construct models closer to the in vivo environment, such as animal models, comprehensively validate the downstream targets of miR-23a-3p and carry out rescue experiments, so as to provide a more solid theoretical basis for the diagnosis and treatment of SP.

## Conclusions

Serum NNT-AS1 is high expression in SP children and exhibits good diagnostic and prognostic value. Moreover, NNT-AS1 modulates LPS-induced damage in human embryonic lung fibroblasts by targeting miR-23a-3p. The results of this study may provide novel insights into the pathological mechanism of SP and identify possible therapeutic targets.

## Supplementary Information

Supplementary Material 1.

Supplementary Material 2: Figure S1. Transfection efficiency was verified by RT‑qPCR. (A). qRT‑PCR analysis of NNT‑AS1 expression in cells transfected with si‑NNT‑AS1. (B). qRT‑PCR analysis of miR‑23a‑3p expression in cells transfected with miR‑23a‑3p inhibitor. Data were analyzed by one‑way ANOVA followed by Tukey’s multiple comparisons test. (***P < 0.001 vs. Control).

Supplementary Material 3. Figure S2. Effects of NNT-AS1 on poly (1:C)-induced injury in MRC-5 cells. Effects of si-NNT-AS1 transfection on NNT-AS1 levels (A), cell viability (B), apoptosis (C), and inflammatory factors (D). Data were analyzed by one‑way ANOVA followed by Tukey’s multiple comparisons test. (***P < 0.001 vs. Control; ##P < 0.01, ###P < 0.001 vs. poly (1:C) + si-NC).

## Data Availability

The datasets used and/or analysed during the current study are available from the corresponding author on reasonable request.
