# Supplementary material for: Expression of long non-coding RNA NNT-AS1 in children with severe pneumonia and its effect on lipopolysaccharide-induced human embryonic lung fibroblast injury
Source: Hereditas. 2026 May 9;163:77. doi: 10.1186/s41065-026-00683-w (PMC13326398; doi:10.1186/s41065-026-00683-w)
Supplement: Supplementary file 1 — Supplementary Material 1. [file 41065_2026_683_MOESM1_ESM.docx]

**Table S1** Primer sequences used for RT-qPCR

| Primer name | Primer sequences (5’-3’) |
| --- | --- |
| NNT-AS1 | F: AGTTCCACCAAGTTTCTTCA |
|  | R: AGGTTTTGCCAGCATAGAC |
| miR-23a-3p | F: GCGATCACATTGCCAGGG |
|  | R: CAGTGCGTGTCGTGGAGT |
| GAPDH | F: AACGGATTTGGTCGTATTGG |
|  | R: TTGATTTTGGAGGGATCTCG |
| U6 | F: GCTTCGGCAGCACATATACTAAAAT |
|  | R: CGCTTCACGAATTTGCGTGTCAT |

Notes: RT-qPCR: real-time quantitative polymerase chain reaction, F: forward, R: reverse.
