# Supplementary figures and images for: Expression of long non-coding RNA NNT-AS1 in children with severe pneumonia and its effect on lipopolysaccharide-induced human embryonic lung fibroblast injury
Source: Hereditas. 2026 May 9;163:77. doi: 10.1186/s41065-026-00683-w (PMC13326398; doi:10.1186/s41065-026-00683-w)

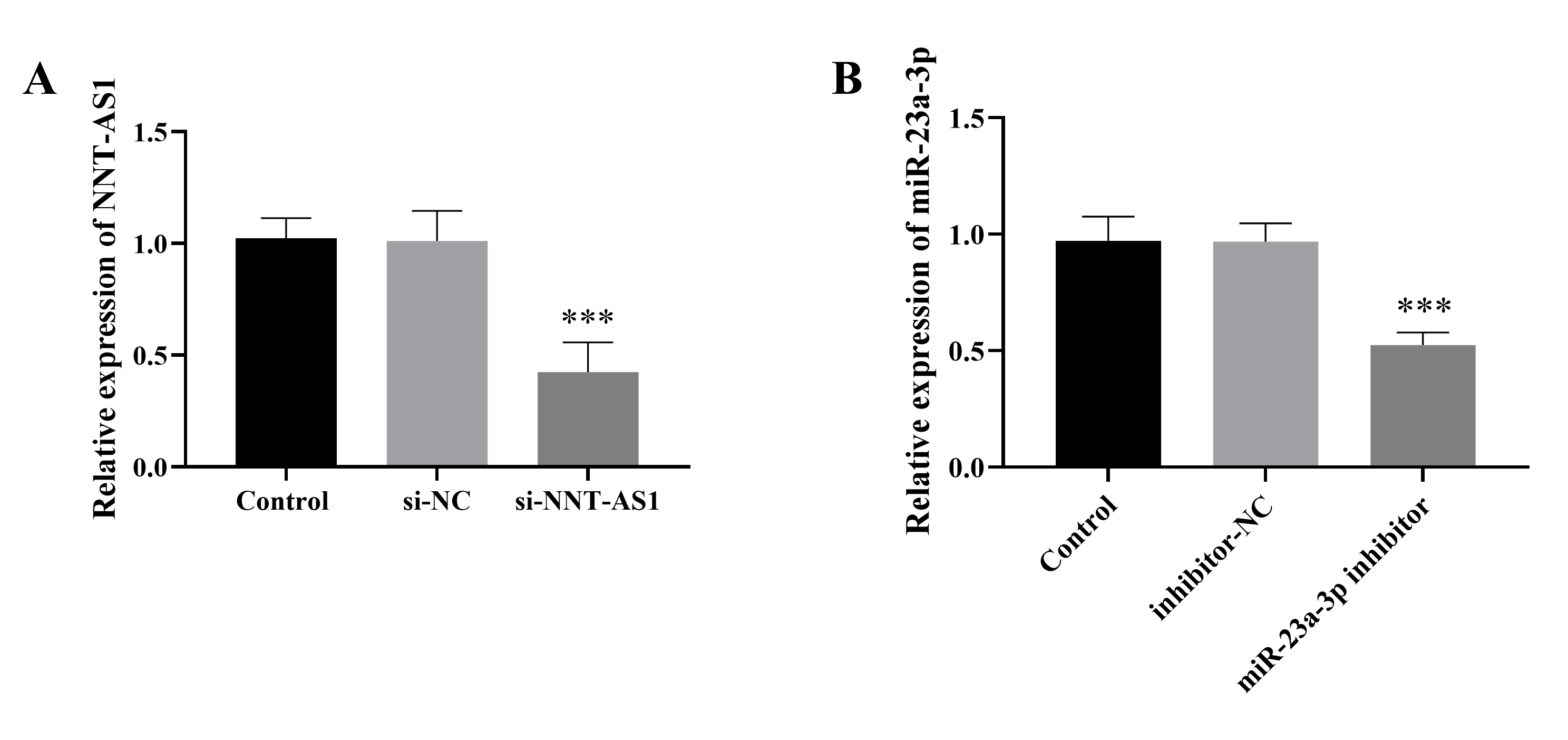

Supplement: Supplementary file 2 — Supplementary Material 2: Figure S1. Transfection efficiency was verified by RT‑qPCR. (A). qRT‑PCR analysis of NNT‑AS1 expression in cells transfected with si‑NNT‑AS1. (B). qRT‑PCR analysis of miR‑23a‑3p expression in cells transfected with miR‑23a‑3p inhibitor. Data were analyzed by one‑way ANOVA followed by Tukey’s multiple comparisons test. (***P < 0.001 vs. Control). [file 41065_2026_683_MOESM2_ESM.tif]

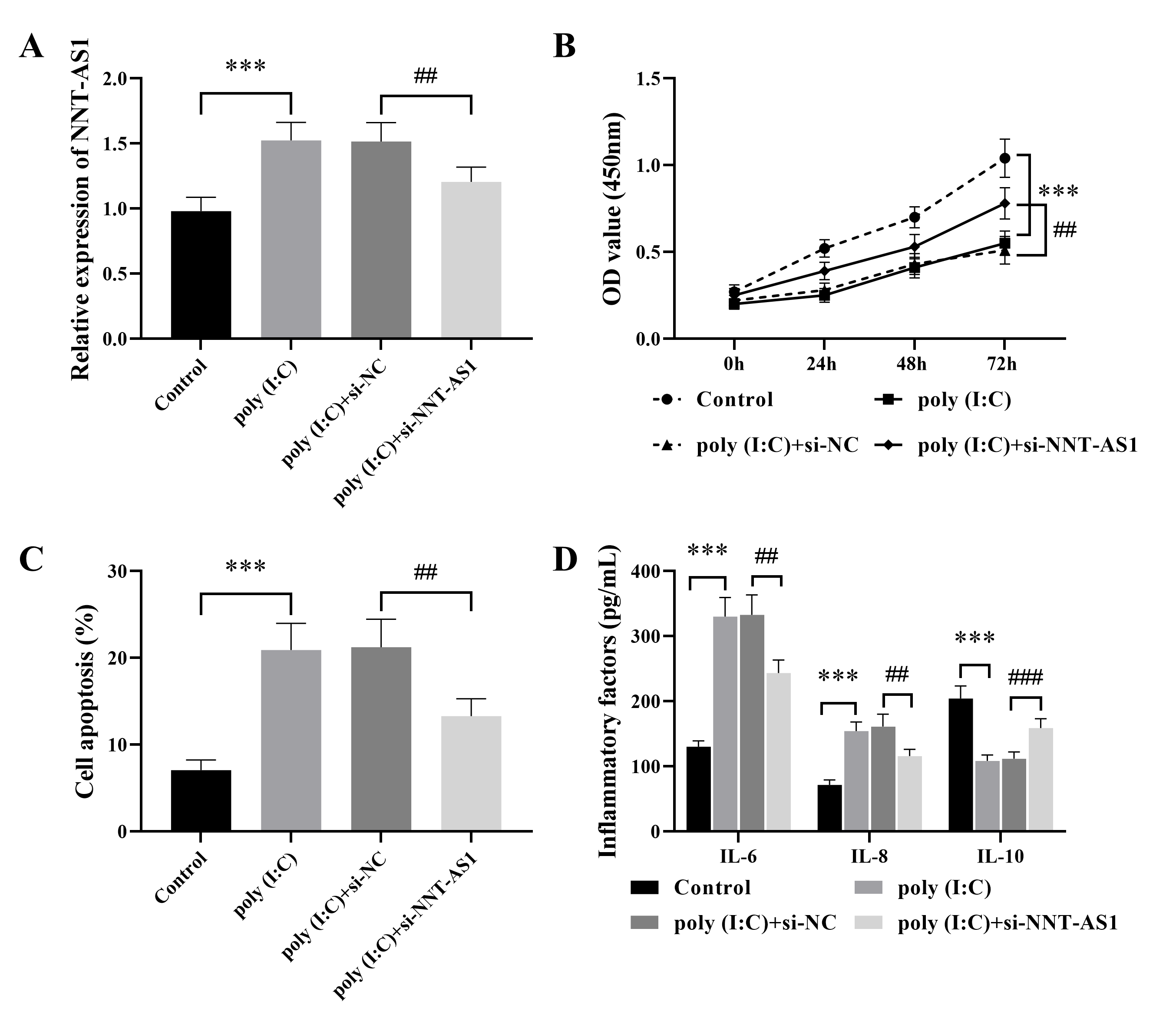

Supplement: Supplementary file 3 — Supplementary Material 3. Figure S2. Effects of NNT-AS1 on poly (1:C)-induced injury in MRC-5 cells. Effects of si-NNT-AS1 transfection on NNT-AS1 levels (A), cell viability (B), apoptosis (C), and inflammatory factors (D). Data were analyzed by one‑way ANOVA followed by Tukey’s multiple comparisons test. (***P < 0.001 vs. Control; ##P < 0.01, ###P < 0.001 vs. poly (1:C) + si-NC). [file 41065_2026_683_MOESM3_ESM.tif]
